# Assessment of Human Skin Gene Expression by Different Blends of Plant Extracts with Implications to Periorbital Skin Aging

**DOI:** 10.3390/ijms19113349

**Published:** 2018-10-26

**Authors:** Jin Namkoong, Dale Kern, Helen E. Knaggs

**Affiliations:** Nu Skin Enterprises, Inc., 75 West Center Street, Provo, UT 84601, USA; jnamkoong@nuskin.com (J.N.); dkern@nuskin.com (D.K.)

**Keywords:** gene expression, skin equivalents, oxidative stress, microRNA, periorbital skin aging, botanicals

## Abstract

Since the skin is the major protective barrier of the body, it is affected by intrinsic and extrinsic factors. Environmental influences such as ultraviolet (UV) irradiation, pollution or dry/cold air are involved in the generation of radical oxygen species (ROS) and impact skin aging and dermal health. Assessment of human skin gene expression and other biomarkers including epigenetic factors are used to evaluate the biological/molecular activities of key compounds in cosmetic formulas. The objective of this study was to quantify human gene expression when epidermal full-thickness skin equivalents were exposed to: (a) a mixture of betaine, pentylene glycol, *Saccharomyces cerevisiae* and *Rhodiola rosea* root extract (BlendE) for antioxidant, skin barrier function and oxidative stress (with hydrogen peroxide challenge); and (b) a mixture of *Narcissus tazetta* bulb extract and *Schisandra chinensis* fruit extract (BlendIP) for various biomarkers and microRNA analysis. For BlendE, several antioxidants, protective oxidative stress biomarkers and many skin barrier function parameters were significantly increased. When BlendE was evaluated, the negative impact of the hydrogen peroxide was significantly reduced for the matrix metalloproteinases (MMP 3 and MMP 12), the skin aging and oxidative stress biomarkers, namely FBN2, ANXA1 and HGF. When BlendIP was tested for cell proliferation and dermal structural components to enhance the integrity of the skin around the eyes: 8 growth factors, 7 signaling, 7 structural/barrier function and 7 oxidative stress biomarkers were significantly increased. Finally, when BlendIP was tested via real-time RT-PCR for microRNA expression: miR-146a, miR-22, miR155, miR16 and miR21 were all significantly increased over control levels. Therefore, human skin gene expression studies are important tools to assess active ingredient compounds such as plant extract blends to advance dermal hypotheses toward validating cosmetic formulations with botanical molecules.

## 1. Introduction

The skin is the major protective barrier of the body and is affected by intrinsic and extrinsic factors. Environmental influences such as ultraviolet (UV) radiation, pollution or dry/cold air are involved in the generation of radical oxygen species (ROS) and impact skin aging and dermal health [1,2,3]. Aging is manifested in various ways with a different time interval for different individuals based on genetics, geographical location, gender, skin type and especially with exposure to the sun and air pollution [2,3,4]. This is especially the case for skin aging [1,2,3,4,5]. Photoaging and ROS production are major contributing causes of skin aging since these factors increase matrix metalloproteinases (MMPs), which break down collagen and elastin, particularly in people who are exposed to the sun, smoke and have unhealthy diets [3,4,5].

Notably, some skin areas behave differently while transitioning between the skin and mucosa, such as the eyelids and lips. Periorbital skin is a special area of the skin that acts as an interface between the facial skin and the eyes. It is often the areas where people notice the first signs of aging [6,7]. The periorbital skin is thinner, and with repetitive muscle movements, the corners of the eye develop lines and wrinkles sooner than other areas of the face, where temporary lines become permanent lines over time [7]. For example, when individuals smile and frown, lines form around the corners of their eyes, mouth and forehead [7,8]. Signs of periorbital aging also include eyelid drooping, dark circles, bags under the eyes or fine lines/wrinkles in this region due to the lack of support from underlying dermal structures that include collagen and elastin fibers [7,9]. The skin around the eye areas is also more sensitive to irritants than other skin regions partially due to a thinner epidermis [9]. Sleep deprivation or water retention are some of the known causes of periorbital aging [9]. Factors that would improve periorbital skin health are antioxidants, anti-inflammatory agents and enhancement of the structural components that make up the skin barrier [6,7,8,9]. In order to develop cosmetic products targeting the face/neck and eye areas, it is important to identify key phytochemicals with antioxidant activities, as well as improvements associated with barrier function [10,11,12,13].

In recent years, increased interests in botanical extracts and phytochemicals (as key compounds) in topical formulations to drive efficacy have been observed [10,11,12,13]. Some of these compounds are known in traditional uses to improve skin health or wound healing, while others are selected due to biological activities on specific areas of interest such as antioxidants, anti-inflammation or skin firmness [10,11,12,13]. For example, the phytochemicals resveratrol and equol have been reported to enhance skin health among various gene expression parameters, including, collagen, elastin and the inhibition of MMPs, ROS and inflammatory biomarkers [11,12,13]. With increasing availabilities of plant extracts, the modulation of skin-related gene biomarkers via gene expression analysis is one of the most beneficial techniques to evaluate the biological actions of botanical blends and phytochemicals [11,12,13]. In this regard, many extracts and compounds are available to be added to cosmetic formulas to improve skin health. For instance, different compounds demonstrate different efficacy in various areas of skin health and have a potential positive impact on the skin. Furthermore, *Rhodiola rosea* root extract includes over 140 compounds, many of which are phytochemicals [14]. This exact is known as an adaptogen, which displays protective and homeostatic roles in the body, as well as anti-fatigue and anti-stress activities [14]. Another example is *Schisandra chinensis*, which is a fruit extract that was used traditionally to fortify the body and reduce fatigue [15]. Thus, these extracts mixed into blends may combine more easily into a cosmetic formulation to further improve their efficacy on skin health.

Additionally, the examination and quantification of epigenetic factors like DNA methylation, histone modification and microRNAs have advanced dermal research and provide valuable tools to determine how gene expression may be modified [16,17]. In fact, microRNAs have been studied in phytochemicals and have been reviewed in reference to skin disorders such as skin cancer and psoriasis [18,19,20].

Thus, the purpose of this study was to determine the efficacy of phytochemical blends: BlendE is a mixture containing *Rhodiola rosea* root extracts, and BlendIP is a mixture containing *Schisandra chinensis* fruit extracts. These blends were tested for various human skin targets associated with antioxidants, extracellular matrix proteins, growth factors, lipid metabolism/transport, dermal structural components, oxidative stress and epigenetic biomarkers that are known to modulate skin aging. This was accomplished by using epidermal full-thickness (EFT) skin equivalents that were exposed to the phytochemical blends in order to quantify human gene or microRNA expression associated with specific targets or categories that modify gene expression in reference to skin aging, especially factors implicating periorbital skin aging. In summary, both plant extract blends tested in the present study demonstrated positive results for various gene expression, biomarker and microRNA parameters suggesting enhancement of skin health and thus implicating potential augmentation of periorbital skin aging.

## 2. Results

### 2.1. BlendE: Modulation of Gene Expression (Antioxidants, Barrier Function, Lipid Metabolism/Transport)

BlendE is a mixture of betaine, pentylene glycol, *Saccharomyces cerevisiae* extract and *Rhodiola rosea* root extract. In order to evaluate whether BlendE has beneficial activities improving skin-associated target genes, BlendE was evaluated on EFT skin equivalents. Medium containing 1.5% of BlendE was applied to skin equivalents from the bottom, as well as from the surface of skin equivalents. After 24 h of treatment, cell viability was evaluated, and RNA was isolated for further assessments. Phytochemical blend concentrations with more than 10% reduction in the viability assays on the EFT cultures compared to the untreated control samples were removed from any further analysis. Real-time RT-PCR was performed on 440 targets known to be important to the skin to evaluate efficacy of BlendE.

**Figure 1:**
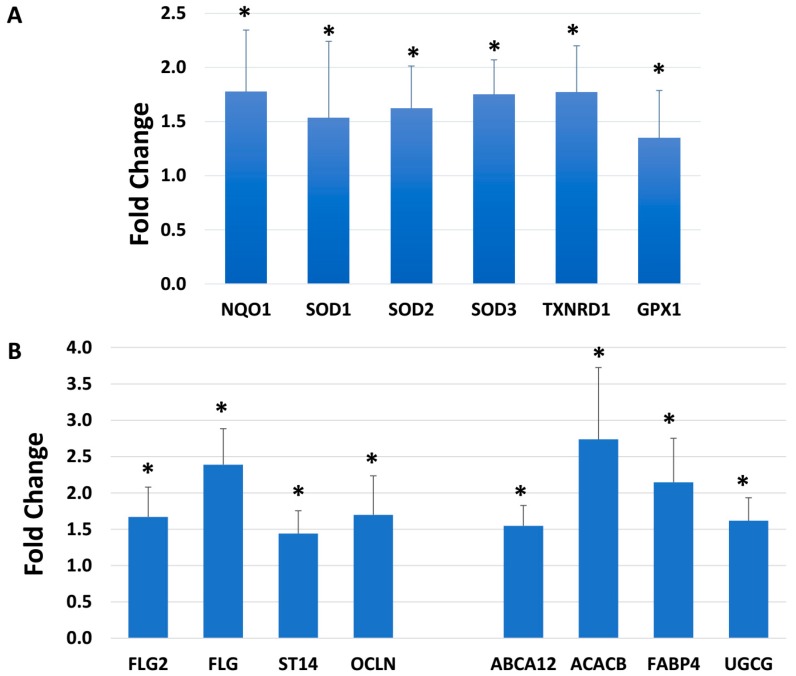
Modulation of gene expression. Skin equivalents were dosed with BlendE for 24 h. Four hundred forty validated Taqman gene expression assays with relevance to the skin structure and function were examined using OpenArray. Unpaired *t*-tests were performed using StatMiner, and only statistically-significant fold changes compared to the untreated samples were shown (*p* ≤ 0.05). Standard deviation is shown as error bars. (**A**) Gene expression changes on selected oxidative stress targets are shown. (**B**) Gene expression changes on selected skin barrier targets are shown. * indicates statistically-significant increase compared to control values.

Among skin-specific genes evaluated, several targets stood out: oxidative stress, skin barrier, fatty acid synthesis and transport, transcription factors and signaling. Selected targets are shown in Figure 1. Superoxide dismutase (SOD) is a well-known antioxidant enzyme that catalyzes the reaction to produce less reactive hydrogen peroxide (H_2_O_2_) from highly reactive superoxide anion. BlendE significantly increased SOD 1, 2 and 3 in a range from 1.5 to 1.7-fold over the control. Furthermore, even though catalase expression did not change with BlendE treatment, GPX1 expression was significantly stimulated, although it displayed less than a 1.5-fold increase. In addition to these antioxidants, NAD(P)H: quinone oxidoreductase (NQO1) and thioredoxin reductase 1 (TXNRD1) were also stimulated, further giving protection from oxidative stress.

For the biomarkers involved in skin integrity and dermal barrier function, BlendE treatment significantly increased the gene expression of filaggrin (FLG) and FLG2 by 1.7 to 2.3-fold, while additionally stimulating occludin (OCLN) and suppressor of tumorigenicity 14 (ST14, also known as matriptase) by 1.4 and 1.7-fold, respectively (Figure 1B).

Finally, lipids and proteins involved in lipid metabolism and transport are also key components of skin barrier function. In this regard, BlendE significantly increased the expression of: (a) ATP-binding cassette sub-family A member 12 (ABCA12) by 1.5-fold, which is important in keratinocyte health/function, (b) acetyl-CoA carboxylase 2 (ACACB) by 2.6-fold, which is an enzyme involved in fatty acid synthesis, (c) fatty acid binding proteins (FABPs) by 2.2-fold, which regulate lipid transport, and (d) UDP-glucose ceramide glucosyltransferase (UGCG) by 1.6-fold, which is a key enzyme in sphingolipid metabolism. Thus, the BlendE results suggest a positive impact on human skin barrier/structural and functional aspects that may help to maintain and improve dermal health.

### 2.2. BlendE: H_2_O_2_ Challenge, Oxidative Stress

Since BlendE was shown to stimulate genes known to protect against oxidative stress, a challenge experiment with H_2_O_2_ was designed. Skin equivalents were challenged with either 5 mM or 10 mM H_2_O_2_ for gene expression changes that mimicked oxidative stress. To evaluate whether BlendE is able to alleviate or abolish the effects of oxidative stress, BlendE was added to H_2_O_2_-challenged skin equivalents. In addition, ascorbic acid was also added to 5 mM H_2_O_2_-challenged skin equivalents. Addition of BlendE or ascorbic acid diminished the impact of H_2_O_2_, lowering the modulation. 

**Figure 2:**
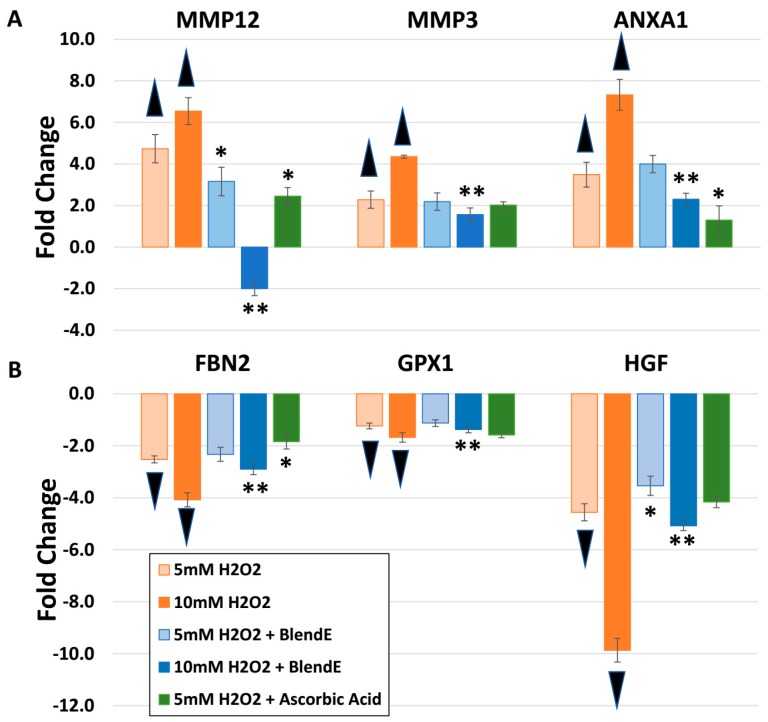
Effects of oxidative stress response on gene expression. Skin equivalents were challenged with 5 mM or 10 mM H_2_O_2_ for 24 h. In addition, known antioxidant compounds, BlendE or ascorbic acids were added to the skin equivalent cultures containing H_2_O_2_ to counteract the effects of hydrogen peroxide. Validated Taqman gene expression assays with relevance to the skin structure and function were examined using OpenArray. Unpaired *t*-tests were performed using StatMiner. Statistically-significant gene expression changes over the untreated are shown (*p* ≤ 0.05). Standard deviation is shown as error bars. Five millimolar H_2_O_2_ is shown in beige, and 10 mM H_2_O_2_ is shown in orange. Five millimolar H_2_O_2_ with BlendE is shown in light blue, and 10 mM H_2_O_2_ with BlendE is shown in blue. Lastly, 5 mM H_2_O_2_ with 50 mg/mL ascorbic acid is shown in green, where ascorbic acid served as the positive control. (**A**) Skin-specific targets with upregulation by H_2_O_2_ are shown. (**B**) Skin-specific targets with down-regulation by H_2_O_2_ are shown. Without H_2_O_2_ challenge, BlendE alone did not modulate MMP12, MMP3, ANXA1, FBN2 and HGF, while GPX1 was stimulated as shown in Figure 1. ▲ indicates statistically-significant increase compared to control levels. ▼ indicates statistically-significant decrease compared to control levels. * indicates statistically-significant difference to 5 mM H_2_O_2_, and ** indicates statistically-significant differences to 10 mM H_2_O_2_ (based on ANOVA, followed by pairwise comparisons, where appropriate).

There are several genes known to be modulated by H_2_O_2_, which represents oxidative stress on skin equivalents. The 5 mM or 10 mM H_2_O_2_ challenge induced MMP12 expression to 4.7 or 6.6-fold, respectively (Figure 2A). Another MMP, MMP3 is also induced by H_2_O_2_ to 2.3 or 4.3-fold. A stress protein known to respond to heat or oxidative stress, annexin A1 (ANXA1) was also significantly induced by 5 mM or 10 mM H_2_O_2_. When BlendE or ascorbic acid was added to H_2_O_2_-challenged skin equivalents, the effect of H_2_O_2_ on MMP12, MMP3 and ANXA1 was diminished to lessen fold changes on these targets, suggesting reduced oxidative stress on skin equivalents.

While MMP3, MMP12 or ANXA1 expression was stimulated by H_2_O_2_ challenge, fibrillin 2 (FBN2), GPX1 and hepatocyte growth factor (HGF) were downregulated by H_2_O_2_ (Figure 2B). FBN2 expression was suppressed over 4-fold by 10 mM H_2_O_2_, which was improved to a 2.9-fold reduction by addition of BlendE. Epithelial cell proliferation inducer, HGF, expression was also suppressed by 5 mM H_2_O_2_ to a 4.6-fold reduction, which was slightly lessened by BlendE to a 3.5-fold reduction. Ascorbic acid did not significantly change. Addition of BlendE to 10 mM H_2_O_2_ reduced the suppression to a 5.1-fold reduction, from a 9.9-fold reduction with H_2_O_2_ alone. GPX1 was also downregulated by 10 mM H_2_O_2_ to a 1.7-fold reduction, while the addition of BlendE reduced to a 1.1-fold reduction, which was a non-significant change from the control sample.

### 2.3. BlendIP: Skin Biomarker (Growth Factors, Signaling, Structural and Oxidative Stress)

**Figure 3:**
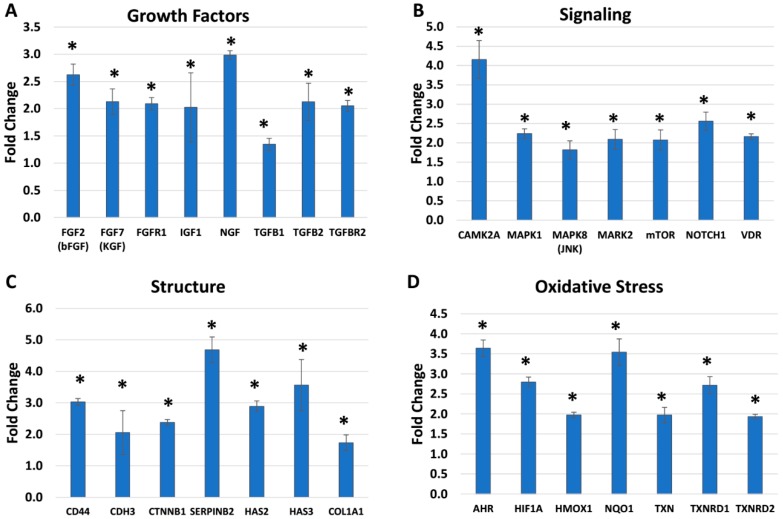
Modulation of gene expression by BlendIP. Skin equivalents were dosed with BlendIP for 24 h. Four hundred forty validated Taqman gene expression assays with relevance to the skin structure and function were examined using OpenArray. Unpaired *t*-tests were performed using StatMiner, and only statistically-significant fold changes compared to the untreated samples were shown (*p* ≤ 0.05). Standard deviation is shown as error bars. (**A**) Gene expression changes on selected growth factors are shown. (**B**) Gene expression changes on selected signaling proteins are shown. (**C**) Gene expression changes on selected skin structure are shown. (**D**). Gene expression changes on selected oxidative stress targets are shown. * indicates statistically-significant increase compared to control values.

Additional biomarkers were evaluated using BlendIP, a mixture of *Narcissus tazetta* bulb extract and *Schisandra chinensis* fruit extract. Compared to BlendE, different skin biomarkers were modulated, while a couple of targets overlapped. For BlendIP, the targets that stood out the most were in growth factors, signaling, skin structure and oxidative stress categories based on different functional or structural components (Figure 3). Growth factors were one of the most highly stimulated targets for BlendIP (Figure 3A). Among selected targets shown, nerve growth factor (NGF), fibroblast growth factor 2 (FGF2, bFGF) or keratinocyte growth factor (KGF, FGF7) were highly stimulated by BlendIP, stimulating targets to 3.0, 2.6 or 2.1-fold, respectively. Growth factors tend to be involved in different signaling pathways, so it was not surprising to see that several signaling molecules were stimulated by BlendIP (Figure 3B). Calcium/calmodulin-dependent protein kinase II (CAMK2A), mitogen-activated protein kinase 1 (MAPK1), mitogen-activated protein kinase 8 (MAPK8, JNK), mitogen-activated protein kinase 2 (MAPK2) or mammalian target of rapamycin (mTOR) are all kinases involved in skin-related signaling pathways. These biomarkers were stimulated from 1.8 to 4.2-fold in this experiment. Even though several signaling molecules are involved in phosphorylation/dephosphorylation for activation/deactivation, which was not measured in this study, changes in expression levels would also impact the pathway.

Skin structure components were also stimulated by BlendIP. For example, a differentiating keratinocyte marker, plasminogen activator inhibitor type 2 (SERPINB2), is upregulated to a 4.7-fold increase. Both hyaluronan synthase 2 (HAS2) and 3 (HAS3) were increased to 2.9 and 3.6-fold, respectively, impacting the skin structure and integrity. Lastly, oxidative stress targets also were modulated by BlendIP. Similar to BlendE, BlendIP also stimulated NQO1 and TXNRD1 to 3.5 and 2.7-fold, respectively. However, there were also additional targets that were stimulated by BlendIP. For example, aryl hydrocarbon receptor (AHR), hypoxia-inducible factor 1 alpha (HIF1A) and heme oxygenase 1 (HMOX1) were stimulated to 3.6, 2.8 and 2.0-fold, respectively.

### 2.4. BlendIP: Epigenetic Modulation

**Figure 4:**
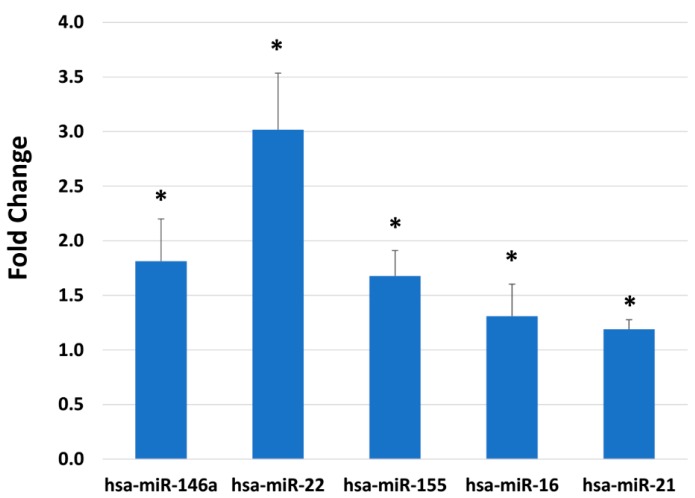
Epigenetic expression modulation. Skin equivalents were dosed with BlendIP for 24 h for microRNA expression. One hundred five validated targets with seven endogenous controls were examined in an OpenArray format. Standard deviation is shown as error bars. Unpaired *t*-tests were performed using StatMiner, and all data shown are statistically-significant from the untreated (*p* ≤ 0.05). Only selected microRNAs are shown. * indicates statistically-significant increase compared to control values.

MicroRNAs are involved in the regulation of many biological processes, such as gene silencing and blocking translation. MicroRNAs are also easily measured using real-time RT-PCR, using microRNA-specific isolation kits and reverse transcription kits. One hundred and five validated microRNAs were assessed after BlendIP treatment for 24 h. Twenty three microRNAs were statistically significantly modulated over the untreated control (*p* ≤ 0.05). Selected targets are shown in Figure 4, which are miR-146a, miR-22, miR-155, miR-16 and miR-21. They were stimulated 1.8, 3.0, 1.7, 1.3 and 1.2-fold, respectively.

## 3. Discussion

In the present study, we investigated the protective effects of two phytochemical blends via skin gene expression and microRNA analysis and showed that the influence of each mixture had a positive impact on skin health. Novel cosmeceuticals from plants have been reported previously that provide anti-aging, anti-wrinkling, anti-oxidant, anti-inflammatory and protection against ROS formation by inhibiting MMPs and stimulating extracellular matrix proteins like collagen and elastin [10,11,12,13,21]. The phytochemical blends containing *Rhodiola rosea* root extracts or *Schisandra chinensis* fruit extracts were examined using various human skin targets associated with antioxidants, extracellular matrix proteins, growth factors, lipid metabolism/transport, dermal structural components, oxidative stress and epigenetic biomarkers that are known to modulate skin aging. The present findings provide new insights into the importance of phytochemical blends since cosmetic products require multiple compounds interacting with each other, as well as within the skin [10,11].

The skin biomarkers were divided into various categories based on their functions and biological activities. While interesting, it is beyond the scope of the present study to report gene characteristics as far as how particular genes may cluster and identify what molecular pathways may be utilized for all the experiments performed. Remarkably, it is known that the fibroblasts in the dermis are responsible for a majority of extracellular matrix proteins and factors involved in maintaining good skin health [22,23]. In fact, a recent report demonstrated that 988 proteins were identified from human dermal fibroblasts where the authors classified these gene products into 13 different major areas along with showing unique patterns of detailed network association and analysis among skin-aging-related proteins [22]. Furthermore, from the human protein atlas, available online, skin-specific gene products have been described as to the cell type and location in the epidermal and dermal layers [23].

In this regard, one of the key categories involved in skin aging is oxidative stress [1,2,3,4,5]. Since skin is the major barrier that protects the body against water loss and external physical, chemical and biological insults, it is known that the concentration or abundance of antioxidants is higher in the epidermis compared to the dermis and has higher levels compared to any internal organs [12,24]. Multiple environmental and intrinsic factors increase ROS within the body including the skin, due to increased production of ROS and reduction in neutralizing enzyme activities [25]. Key enzymes in the neutralization of ROS are SODs. In humans, three SODs have been identified, and they function at different locations: SOD1 is present in the cytoplasm and nucleus; SOD2 functions in the mitochondria; and SOD3 functions in extracellular spaces [26]. SODs represent the important first line of defense, activated when inflammation is induced by extrinsic factors such as UV irradiation and pollution [12]. BlendE stimulated all three SOD genes in their expression to bolster the antioxidant system in the skin. The present SOD results are similar to those previously reported by our laboratory and that of other investigators when phytochemicals were studied [12,13,27]. In examining the effects of H_2_O_2_ produced during the oxidative stress reaction, this requires further detoxification, which could be catalyzed by catalase or GPX1, which was also stimulated by BlendE [28]. In addition to BlendE, BlendIP also stimulated oxidative stress target genes, such as HMOX1 and NQO1. In this pathway, the aryl hydrocarbon receptor (AHR) was increased, which in turn is known to activate the cytochrome P450 CYP1A1 gene involved in oxidative stress, but certain phytochemicals are known to induce the antioxidant enzymes such as NQO1, as seen in the present study [29]. To complete this mechanism, both thioredoxin (TXN) and TXNRD were increased (see Figure 3D), which are involved in oxidoreductase pathways associated with protein repair and redox homeostasis, while the hypoxia inducible factor 1 alpha (HIF1A; see Figure 3D) is known to be regulated by phytochemicals such as epigallocatechin-3-gallate, which is the main polyphenol component of green tea [30].

The periorbital skin region is thinner than other parts of the body. In order to strengthen the integrity of the skin around the eyes, it is important to stimulate cell proliferation and boost the structural dermal components, especially with aging [7,8,9]. This is especially the case when loss of firmness of the skin is instigated by oxidative stress [12,31]. In the present study, using a H_2_O_2_ challenge as the oxidative stressor in the human skin equivalents, several targets like MMPs and FBN2 were shown to reverse the potentially damaging effects of oxidative stress by BlendE, demonstrating the potential improvements to the skin integrity. In addition, BlendIP stimulated several growth factors, signaling molecules and skin structural protein expressions, to boost skin health. Again, these results are somewhat similar to those reported by our laboratory and by others where different phytochemicals were tested such as equol and resveratrol [13,27].

In examining the protective barrier of the skin, it is important to remember that the skin guards the internal organs from outside insults and maintains the barrier for internal influences, while it dynamically sheds the outermost epidermal layers continuously [12,22,31]. In order to properly function as a barrier, four major components need to work together [32]. They are the intercellular lipid layers, the corneocyte lipid envelope, the cornified cell envelope and keratin and filaggrin and their breakdown components [32]. The most well-known skin barrier protein is FLG, whose loss-of-function in mutation studies have been linked to a permeability barrier abnormality and atopic dermatitis [33]. Since BlendE stimulated FLG and FLG2, these results suggest that skin barrier function would be enhanced. Lastly, BlendIP also increased the growth factors, like FGF, IGF1, NGF and TGFBs, and signaling and structural genes, which confirms the results of previous journal reports and expands our understanding of how phytochemicals via human skin gene expression studies enhance dermal health [13,27].

Other key components of skin barrier function include lipids and proteins involved in lipid metabolism and transport. For example, skin biomarker ABCA12 transports lipids in keratinocytes, especially in lamellar granules [34]. Mutations in ABCA12 have been shown to lead to congenital ichthyoses, while increased ABCA12 expression stimulates epidermal lipid and lamellar bodies [34]. The results of the present study demonstrated an upregulation of ABCA12 expression by BlendE, which supports the notion that improved keratinocyte differentiation may result. Another skin biomarker is UGCG, which can cause ichthyosis when levels are decreased, however, the BlendE treatment stimulated UGCG in this study [32]. Finally, when skin genes involved in fatty acid synthesis and fatty acid binding proteins (FABPs) that regulate lipid transport were examined [35,36], the fatty acid synthesis enzyme ACACB was increased by the BlendE treatment, as well as the FABPs. This suggests that the BlendE effects may reduce trans-epidermal water loss (TEWL) due to an increase in skin barrier function along with enhancing lipid transport among the epidermal layers [35,36]. Notably, these skin components are critical in maintaining skin barrier function, cellular transport of lipid molecules and modulating the optimal levels of especially keratinocyte differentiation in the epidermis [35,36].

Epigenetics in recent years has attracted research awareness and provided an additional level of how gene expression is regulated [37]. In summary, epigenetics is the inheritance of changes in gene function without any changes in the genetic sequences [37]. This means without any specific changes to nucleotide sequences, there are measurable differences in gene expression. For example, aging and chronic sun exposure cause distinct epigenetic changes in human skin, meaning in this case, aging is a phenotypic change, not a genetic change [16]. Furthermore, in 2018, the epigenetics of skin cancer was reported where bioactive phytochemicals served as intervention treatments [18]. Thus, epigenetics plays a critical role in the expression of genetic traits [16]. The most well-known epigenetic modifications are DNA methylation, histone modifications and regulatory RNAs. Among these, microRNA arrays are broadly used in research, including evaluating topical ingredients by utilizing real-time RT-PCR [17,19,20]. Finally, microRNAs in human skin aging have been reviewed recently along with the advances and applications of microRNAs as therapies for skin diseases like psoriasis [19,38,39].

Global microRNA expression is upregulated during aging [38,40]. MicroRNAs are 19 to 24-nucleotide non-coding RNA sequences that negatively regulate gene expression by RNA transcript degradation or inhibition of translation [41]. In addition, microRNA can activate translation in quiescent cells [41]. BlendIP modulated various microRNA expression, selected based on previous reports and targets. Among the microRNAs tested, miR-22 was most highly modulated by BlendIP. miR-22 was identified to be upregulated when human keratinocytes were exposed to UV irradiation, to stimulate cell survival affecting phosphatase and tensin homolog (PTEN) expression [42]. miR-146a is also stimulated by BlendIP. miR-146a is an important inflammatory regulator, whose expression is partially controlled by nuclear factor-kappa B (NF-κB) [43]. As a feedback mechanism, miR-146a also inhibits the upstream of NF-κB signaling pathways [43]. It has been shown that the natural phytochemical troxerutin (a bioflavonoid of rutin) can alter microRNA expression profiles that are involved in its protective effects against UV-B radiation in normal human dermal fibroblasts [44]. Unfortunately, in this previous study, it is not possible to compare the results to the present study due to differences in experimental design [44]. Even though some targets of microRNAs are known, specific targets of various microRNAs and how they impact the overall changes that would occur on the cell are still unknown in reference to human skin health.

The environment influences microRNA expression, as well. There were several published studies examining environmental exposure in utero modulating microRNAs [45]. Comparing microRNAs from placentas procured during delivery presented downregulation of miR-146a, miR-16 and miR-21, when the mother had a history of smoking during pregnancy [45]. It is well known that smoking has a large negative impact on skin health [46,47]. Furthermore, it is interesting to find in this study that all three microRNAs identified were upregulated by BlendIP. Many studies that have examined different microRNAs from the skin showed that microRNAs are associated with skin cancers or skin diseases [18,19,20,38,39]. Finally, it is also important to understand how microRNAs keep the skin healthy, to prevent skin aging.

In summary, skin equivalents are a valuable tool to evaluate how human skin target genes are modulated, when different compounds are applied to the skin. In this perspective, botanicals such as phytochemicals and various extracts and compounds are known to improve skin dermal health [2,10,11,12,13,21,27,30]. Utilizing human skin gene expression studies in the present study, two phytochemical blends (BlendE and BlendIP) were formulated into topical applications to determine their influence on various skin attributes. Both topical blend formulations were able to improve several skin attributes, particularly related to oxidative stress and periorbital skin aging. The present gene analysis that used in vitro assessments is part of the critical protocol to advance dermal hypotheses and work towards additional in vitro and in vivo investigations to validate cosmetic formulations with botanical extracts. However, further research study is necessary to elucidate the gene/molecular/biochemical pathways and interactions of the component compounds/enzymes in order to understand the complex mechanisms that lead to dermal homeostasis and improved skin health, especially with aging.

## 4. Materials and Methods

### 4.1. Cell Culture 

Human skin equivalents are a useful tool to assess and evaluate different compounds, prior to formulation and in vivo clinical evaluation. Skin equivalents are an artificial skin model made with more than one cell types, which behaves like the human skin. It does not contain all available cells present in human skin like immune cells, but it usually comprises of keratinocytes and fibroblasts. Keratinocytes and fibroblasts are very different cell types with different behavior and functions. Keratinocytes are epithelial cells, which differentiate to form a barrier, whereas fibroblasts form a structural component by making extracellular matrix (ECM). Skin equivalents are used to assess changes that occur when keratinocytes and fibroblasts are cultured together in three-dimensional constructs.

In order to evaluate different cosmetic ingredients on genetic and epigenetic modulations, EFT human skin equivalents were obtained from MatTek (EFT-400, Ashland, MA, USA). These human skin equivalents were made from normal human fibroblasts and normal human keratinocytes from neonatal sources, followed by exposure to the air-liquid interface. Keratinocytes proliferate and differentiate to form multiple cell layers with the stratum corneum, mimicking human skin. Skin equivalents go through quality assurance processes at the manufacturer site. After equilibrating the skin equivalents according to the manufacturer’s protocol, they were dosed with various concentrations of different compounds in four to six replicates using the supplied media.

BlendE is a mixture of betaine, pentylene glycol, *Saccharomyces cerevisiae* extract and *Rhodiola rosea* root extract. BlendIP is a mixture of *Narcissus tazetta* bulb extract and *Schisandra chinensis* fruit extract. Hydrogen peroxide was used to induce oxidative stress, and ascorbic acid was used as an antioxidant. One hundred microliters of diluted compounds were applied to the center of each skin equivalent, and a sterile glass spreader was used to spread evenly. In addition, media containing experimental compounds were fed to the skin equivalents from the bottom up. After a 24-h incubation period, the skin equivalents were harvested for cell viability and genetic/epigenetic analysis. Phytochemical blend concentrations with more than 10% reduction in the viability assays on the EFT cultures compared to the untreated control samples were removed from any further analysis.

### 4.2. RNA Isolation and Real-Time Reverse Transcriptase Polymerase Chain Reaction

In order to isolate RNA, skin equivalents are first rinsed with sterile PBS and harvested in RNAlater (Qiagen, Valencia, CA, USA) using a Maxwell 16 LEV simply RNA Tissue kits (Promega, Madison, WI, USA) according to the manufacturer’s protocol. For microRNA analysis, RNA was isolated using miRNeasy Mini Kits (Qiagen) according to the manufacturer’s protocol. Isolated RNAs from both kits were evaluated for concentration and quality using a NanoDrop 2000 (Thermo Fisher Scientific, Waltham, MA, USA). cDNA was synthesized using High Capacity DNA Synthesis Kits (Thermo Fisher Scientific) for mRNAs and Taqman microRNA Reverse Transcription kits (Thermo Fisher Scientific), and pre-amplified for 12 cycles using a Taqman Custom microRNA Preamplification Primer Pool (Thermo Fisher Scientific) and Taqman PreAmplification Master Mix (Thermo Fisher Scientific) were used for microRNAs.

Validated Taqman gene expression assays with endogenous control genes or Genemarkers’ microRNA Skin Panel (Kalamanzoo, MI, USA) were put together in custom OpenArray (Thermo Fisher Scientific) formats and evaluated using the QuantStudio 12K Flex instrument (Thermo Fisher Scientific). For the gene expression analysis using BlendE, hypoxanthine phosphoribosyltransferase 1 (HPRT1) was selected as the endogenous control gene, and for the gene expression analysis using BlendIP, glyceraldehyde 3-phosphate dehydrogenase (GAPDH) served as the endogenous control gene. For microRNA experiment, RNU48 served as the endogenous control.

### 4.3. Data and Statistical Analysis

Real-Time RT-PCR data were analyzed using RealTime StatMiner software v4.2 (Thermo Fisher Scientific) for statistical analysis using the relative quantitation (RQ) method. The cycle threshold (CT) value of the target was normalized to the CT value of a selected endogenous control. RQ value was calculated and converted to linear fold changes. Unpaired *t*-tests were performed, and a *p*-value of less than or equal to 0.05 was reported as statistically significant results (*p* ≤ 0.05), as reported previously [27]. For comparison between groups of the H_2_O_2_ challenge, data were analyzed using analysis of variance (ANOVA) followed by Tukey’s honestly significant difference (HSD) post-hoc tests [48]. Treatment groups were compared to the same H_2_O_2_ concentration groups, and a *p*-value of less than or equal to 0.05 was reported as statistically significant results (*p* ≤ 0.05). All results were expressed as mean ± standard deviation.

## Abbreviations

ABCA12	ATP-binding cassette sub-family A member 12
ACACB	acetyl-CoA carboxylase 2
AHR	aryl hydrocarbon receptor
ANXA1	annexin A1
CAMPK2A	calcium/calmodulin dependent protein kinase II
CD	cell adhesion
CDH3	cadherin
COL1A1	collagen type 1 alpha 1
CTNNB1	beta-catenin
ECM	extracellular matrix
EFT	epidermal full-thickness
FABP	fatty acid binding protein
FBN	fibrillin
FGF	fibroblast growth factor
FLG	filaggrin
GPX1	glutathione peroxidase 1
H_2_O_2_	hydrogen peroxide
HAS	hyaluronan synthase
HGF	hepatocyte growth factor
HIF1A	hypoxia-inducible factor 1 alpha
HMOX1	heme oxygenase 1
IGF	insulin growth factor
MAPK	mitogen-activated protein kinase
MARK2	serine/threonine-protein kinase
MMP	matrix metalloprotease
mTOR	mechanistic target of rapamycin kinase
NGF	nerve growth factor
NOTCH1	transmembrane protein 1
NQO1	NAD(P)H: quinone oxidoreductase
OCLN	occludin
ROS	radical oxygen species
SERPINB2	plasminogen activator inhibitor type 2
SOD	superoxide dismutase
ST14	suppression of tumorigenicity 14
TEWL	trans-epidermal water loss
TGF	transforming growth factor
TXN	thioredoxin
TXNRD1	thioredoxin reductase 1
VDR	vitamin D receptor
UGCG	UDP-glucose ceramide glucosyltransferase
UV	ultraviolet
